# Effects of administration of dexmedetomidine on inflammatory responses and severity in severe septic patients

**DOI:** 10.1186/cc14567

**Published:** 2015-03-16

**Authors:** T Taniguchi, M Okajima, K Sato, T Noda

**Affiliations:** 1 Kanazawa University, Kanazawa, Japan

## Introduction

Recently, several animal studies observed that dexmedetomidine (DEX), a new sedative and α_2_-adrenoceptor agonist, inhibited the inflammatory responses [1-3]. Moreover, DEX was reported to have anti-inflammatory effects in patients [4,5]. However, these studies were the small-sized studies and there are few studies about the effects of long-term administration of DEX in severe septic patients. The present study evaluated the effects of long-term administration of DEX on inflammatory responses and severity in severe septic patients. We hypothesize that the administration of DEX has beneficial effects for severe septic patients.

## Methods

In 66 patients (M/F 44/22, mean age 66 years) with severe sepsis, who were administered propofol (0.5 to 4.0 mg/kg/hour) only for sedation, 42 patients (M/F 28/14, mean age 67 years) were administered DEX (0.2 to 0.7 μg/kg/hour) for more than 24 hours in addition to propofol (DEX group). Twenty-four patients were not administered DEX (Control group). Primary outcome were changes in inflammatory responses at 48 hours after the administration of DEX or none, and secondary outcomes were changes in APACHE II and SOFA scores at 48 hours after the administration of DEX or none.

## Results

The administration of DEX occurred for a mean 130 hours (24 to 433 hours) in the DEX group. White blood cell counts, C-reactive protein (CRP) and procalcitonin (PCT) in both groups significantly decreased after the administration of DEX or none. However, CRP and PCT in the DEX group were significantly lower than those in the control group: CRP 7.7 (5.0) versus 13.6 (7.9) mg/dl; *P *< 0.05, PCT 7.6 (11.7) versus 18.6 (11.6) ng/ml; *P *< 0.05, mean (SD). APACHE II and SOFA scores in both groups decreased after the administration of DEX or none, but APACHE II and SOFA scores in the DEX group were lower than those in the control group: APACHE II 10.8 (4.8) versus 15.2 (5.1); *P *< 0.05, SOFA 3.6 (2.0) versus 5.8 (2.9); *P *< 0.05, mean (SD).

## Conclusion

In the present study, the long-term administration of DEX has beneficial effects of inflammatory responses and severity for severe septic patients.
